# Reducing the γ′-Particle Size in CMSX-4 for Membrane Development

**DOI:** 10.3390/ma15041320

**Published:** 2022-02-10

**Authors:** Janik Marius Lück, Joachim Rösler

**Affiliations:** Institut für Werkstoffe, Technische Universität Braunschweig, Langer Kamp 8, 38106 Braunschweig, Germany; j.roesler@tu-braunschweig.de

**Keywords:** γ′-precipitates, growth of γ′, CMSX-4, superalloy membranes, heat treatment

## Abstract

Colloidal emulsions for lipophilic drugs can be fabricated using premix membrane emulsification. The state of the art is the application of membranes made from, for example, polycarbonate or polyester, which, however, are prone to fouling and cause waste, due to the low number of cycles. With the use of metallic membranes made from the nickel based single crystalline superalloy CMSX-4, these key disadvantages are eliminated. However, instead, the pore size and the resulting droplet size distribution need to be adjusted and improved. This can be realized by tailoring the size of the γ′-particles, which is controllable by the time and temperature used during precipitation heat treatment and the quenching method after homogenization heat treatment. Therefore, we utilized different heat treatment protocols, varying the cooling rate (water quenching and air cooling) after homogenization heat treatment and the holding time and temperature during precipitation heat treatment. Then, we investigated the γ/γ′-microstructure, including the γ′-morphology and γ′-particle size. We show that water quenching has a significant impact on the γ/γ′-microstructure and often leads to irregular-shaped and poorly aligned γ′-particles after precipitation heat treatment. In comparison, air cooling, followed by a subsequent precipitation heat treatment, results in well-aligned and cubic shaped γ′-particles and is, therefore, favorable for membrane fabrication. A reduction in precipitation temperature leads to morphology changes to the γ′-particles. A reduction of the holding time during precipitation heat treatment diminishes the γ′-particle growth, resulting in smaller γ′-particles. Additionally, a suitable heat treatment protocol for membrane fabrication was identified with a γ′-edge length of 224 ± 52 nm and well-aligned, cubic shaped γ′-particles.

## 1. Introduction

The production of nanodrug delivery systems can be realized by different processes. Commonly, high-pressure homogenization is used to manufacture nanoemulsions that carry lipophilic drugs for intravenous, personal, and dermal administration [1]. One key disadvantage is the principle of size reduction, which is based on cavitation, high shear stresses, and the collision of particles, resulting in increased temperatures [1,2]. Sensitive components may suffer under these conditions, leading to a loss of functional properties [3]. A promising alternative technique is premix membrane emulsification (premix ME), which can be used to produce colloidal emulsions as carrier systems for lipophilic drugs. Droplet sizes smaller than 500 nm and a narrow particle size distribution enable the drug carrier system to be administered directly into the bloodstream [4]. The premix ME uses a porous membrane. By pushing the pre-mixed emulsion with large droplet sizes through the membrane, the droplets become disrupted by the pores of the membrane into finer droplets. The process leads to a narrow droplet size distribution and smaller mean droplet size, with a lower use of energy [1,5]. Despite the advantages of premix ME, the materials (i.e., polycarbonate or polyester) and membranes used are fragile and prone to fouling, resulting in short operating times and high disposal rates [1]. As a possibility to overcome these shortcomings, the applicability of metallic, nanoporous membranes made from the nickel-based single crystalline superalloy CMSX-4^®^ [6] was proven by [1]. Several studies, including by [7,8,9,10,11,12,13], developed and investigated the production process, including mechanical characterization. In general, the fabrication of a porous nickel-based superalloy membrane consists of three fundamental steps: heat treatment, directional coarsening, and electrochemical phase extraction [1,12,13]. The heat treatment is subdivided into homogenization and precipitation heat treatment. While homogenization should provide a uniform distribution of the alloying elements, aging is used to precipitate the coherent Ni_3_ (Al, Ti, Ta) γ′-phase from the γ-matrix. Due to the small lattice parameter misfit δ between the two phases, which results in a low interfacial energy, the γ′-phase shows good thermal long-term stability. Since the misfit δ is temperature dependent and the size and shape of the γ′-particles is a function of the misfit δ, the precipitation temperature has a significant influence on the final size and shape of the γ′-particles [14,15,16]. In addition to the misfit δ, the cooling rate after homogenization is an influencing factor, since it affects the nucleation rate and, thus, the initial size and distribution of the γ′-particles [17,18]. Following the precipitation process, a thermo-mechanical treatment is used to directionally coarsen the γ′-particles, until the two phases are themselves connected and interpenetrate each other. In the third step, the electrochemical phase extraction, either the γ- or γ′-phase can be selectively dissolved, due to their different electrochemical potentials. After the phase extraction, the resulting porous structure provides pores in the range of a few hundred nanometers [1,7,13].

Since the membrane pore size correlates with the droplet size and distribution, one main goal is to reduce the size of the pores in nickel-based superalloy membranes, to further improve the emulsification performance [3]. The initial size of the γ′-particles after aging may be one of the key parameters for reducing the final size of the membrane pores. Rösler et al. [8] showed the influence of the cooling rate after the homogenization treatment, leading to finer pores as the cooling rate is increased in several steps, from 0.2 K/min to air cooling. Therefore, we examined three approaches in this research:

1. Water quenching instead of air cooling after homogenization, leading to a higher nucleation rate and a higher amount of viable seeds and, thus, possibly to smaller γ′-particles after precipitation heat treatment.
2. The reduction of the precipitation time, in order to diminish the growth of the γ/γ′-microstructure, leading to smaller γ′-precipitates and finer γ-ligaments between them.
3. The reduction of the precipitation temperature. Similarly to reduced precipitation time, this measure is expected to diminish the growth of the γ/γ′-microstructure. However, further effects are likely, as it may also influence phase fractions and the misfit δ.

After explaining materials and methods in Section 2, we will address the influence of the cooling rate in Section 3.1. The effects of precipitation time and temperature are then shown in Section 3.2 and Section 3.3, respectively. Finally, the results are discussed and summarized in Section 4 and Section 5.

## 2. Materials and Methods

To investigate the influence of water quenching on the particle shape and size, the single crystalline nickel-based superalloy CMSX-4 was used. Cubic samples (20 mm × 20 mm × 20 mm) were cut from a single crystal plate and then homogenized. The long axis of the plate deviated by 6–10° from the [001]-orientation, as measured by electron backscattered diffraction (EBSD) using a scanning electron microscope (FEI Helios NanoLab 650). After homogenization heat treatment (HT), samples were either air cooled (AC) or water quenched (WQ) and cut into eight identically sized cubes for additional precipitation heat treatment. To compare the effect of water quenching and air cooling after homogenization, all subsequent heat treatments were carried out on both cooling variants. After precipitation heat treatment, samples were either air cooled to room temperature or furnace cooled (FC) with 50 K/h, until they reached 550 °C. The heat treatment process, including homogenization and precipitation, is summarized in Table 1. Samples were cut in half parallel to the [001]-orientation and prepared by grinding and polishing followed by etching with molybdic acid for 4 s. SEM images were taken with a ZEISS LEO 1550 Gemini scanning electron microscope. The γ′-edge lengths parallel to the [001]-orientation were measured with ImageJ, using the built-in length measuring tool with a total amount of 120 random γ′-particles from three different images taken at random locations. Then the mean edge length and the standard deviation were calculated. The γ′-volume fraction was measured manually using the ImageJ Point Analysis plug-in [19]. As the measurement was performed manually, only three images were used to determine the mean γ′-volume fraction. This must be taken into account when considering the standard deviations.

## 3. Results

### 3.1. Influence of Water Quenching after Homogenization on the Microstructure and γ′-Particle Size

**Figure 1:**
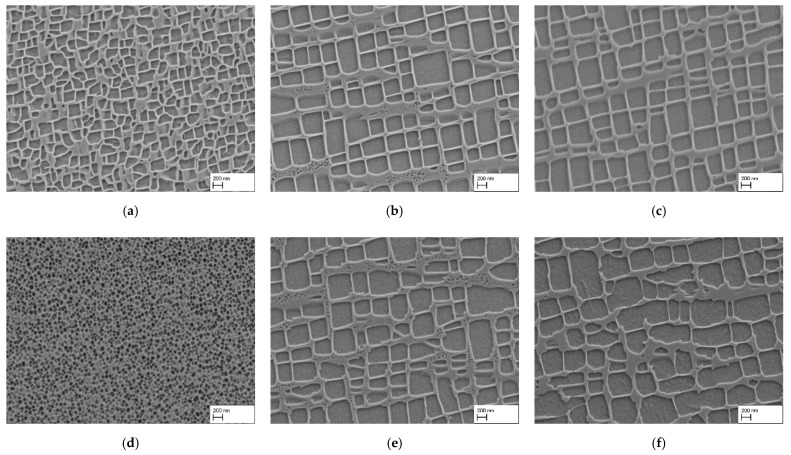
γ/γ′-microstructure after homogenization and precipitation heat treatments: (**a**) HT/AC, (**b**) HT/AC + 1140 °C/6 h/AC, (**c**) HT/AC + 1140 °C/6 h/50 K/h cooling until 550 °C, (**d**) HT/WQ, (**e**) HT/WQ + 1140 °C/6 h/AC, (**f**) HT/WQ + 1140 °C/6 h/50 K/h cooling until 550 °C.

Figure 1 summarizes the investigation of the impact of water quenching compared to air cooling after the homogenization heat treatment and the resulting consequences for the γ/γ′-microstructure after subsequent precipitation heat treatments. Figure 1a shows the microstructure after air cooling from homogenization heat treatment. The visible γ′-precipitates indicate a distribution of different forms, such as rectangles, triangles, and polygons. After precipitation heat treatment for 6 h at 1140 °C, followed by air cooling, the γ′-precipitates are very well aligned, with a narrower size distribution (Figure 1b). However, some bigger and smaller γ′-precipitates are clearly present. Furthermore, the γ-matrix phase contains visible secondary γ′-precipitates. Apparently, supersaturation of the γ-matrix channels with the γ′-forming elements during cooling from 1140 °C led to the formation of these fine precipitates. However, if furnace cooling was selected, secondary γ′-particles are essentially absent (Figure 1c), while the shape and alignment of the primary ones remain unaltered. Apparently, the γ′-forming elements have enough time to diffuse towards the existing γ′-precipitates, so that supersaturation of the γ-matrix diminishes and nucleation of new γ′-particles is hindered. Figure 1d shows the microstructure after water quenching from homogenization heat treatment. Compared to (a), the number of γ′-precipitates is significantly higher, and the size is distinctly smaller. However, contrary to the previously proposed hypothesis, the microstructure in (e) after precipitation heat treatment at 1140 °C for 6 h did not lead to smaller γ′-precipitates. They are also not well aligned and show some curved particle edges with undefined forms. Furnace cooling with 50 K/h instead of air cooling significantly impaired the microstructure, resulting in a frayed matrix phase, while the shape of the precipitates becomes more irregular (f).

Additionally, the γ′-volume fraction and γ′-edge length are summarized in Table 2. The results show that the γ′-edge length is smaller for the air cooled samples compared to those water quenched. The smallest mean γ′-edge length was measured for the AC sample, with a following annealing at 1140 °C/6 h/AC. It is about 345 ± 87 nm, with a γ′-volume fraction of 59.6 ± 1.9%. In comparison, the water quenched sample shows a mean γ′-edge length of 445 ± 182 nm and a γ′-volume fraction of 57.3 ± 2.3% after the same precipitation heat treatment. With furnace cooling the mean γ′-edge length and the mean γ′-volume fraction increased in both cooling variants (AC: 64.4 ± 2.4% and 370 ± 107 nm; WQ: 69.1 ± 2.5% and 566 ± 373 nm). It is noticeable, that the standard deviation of the mean γ′-edge length of the water quenched sample is comparatively high.

All in all, this comparison shows that water quenching after homogenization heat treatment affects the γ/γ′-microstructure in several respects. First, the γ′-particle size is higher; and, second, the particle shape is more irregular when water quenching is used instead of air cooling. Furthermore, it is noted that the γ′-volume fraction increases when applying furnace cooling with a cooling rate of 50 K/h.

In all considered heat treatments, the microstructures were not favorable for the development of membranes with decreased pore size, because of the relatively large size of the γ′-particles and the irregular shape in case of water quenching. It is noted that the formation of secondary γ′ can be hindered by furnace cooling; however, the mean γ′-edge length is increased, leading to bigger γ′-particles.

The main goal was to reduce the edge length of the γ′-precipitates, which was about 345 ± 87 nm for the heat treatment at 1140 °C for 6 h, in the following, referred to as standard heat treatment. Moving on from this size, the further investigation was focused on size reduction using lower precipitation temperatures and reduced holding times.

### 3.2. Reduction of the Precipitation Time

**Figure 2:**
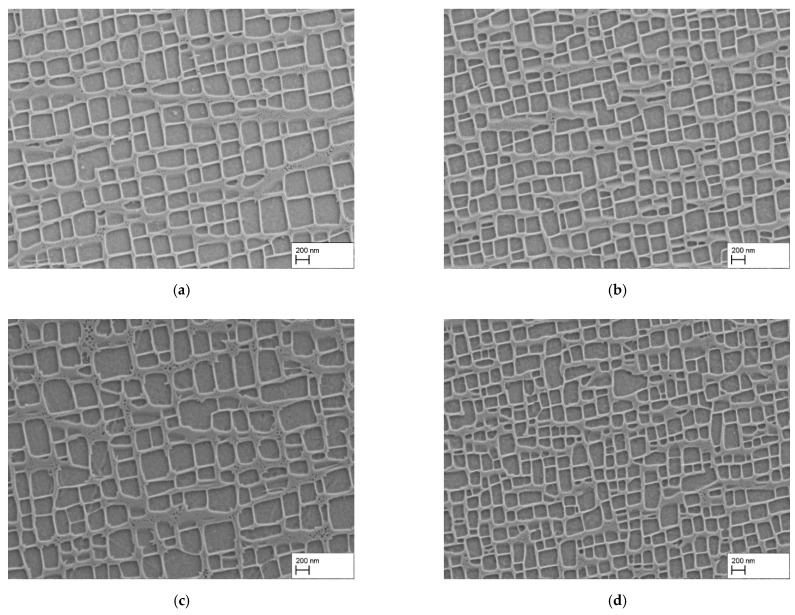
γ/γ′-microstructure after homogenization and precipitation heat treatments: (**a**) HT/AC + 1140 °C/2 h/AC, (**b**) HT/AC + 1140 °C/30 min/AC, (**c**) HT/WQ + 1140 °C/2 h/AC, (**d**) HT/WQ + 1140 °C/30 min/AC.

The first attempt to reduce the size of γ′-particles and inhibit the γ-ligament growth at the same time, was to reduce the holding time during precipitation heat treatment. For completeness, water quenched and air cooled samples were also compared. Figure 2 summarizes the results. Figure 2a,b show the γ/γ′-microstructure of air cooled samples with a holding times of (a) 2 h and (b) 30 min at 1140 °C. It is noticeable that the size of the γ′-particles is smaller for both heat treatments compared to the standard heat treatment of 6 h. In addition, there are differences between the 2 h and the 30 min heat treatments. While the γ/γ′-microstructure is finer in the 30 min sample, the alignment of the γ′-particles along their side faces is more pronounced after the 2 h heat treatment. The edginess appears to be quite similar. With water quenching after the homogenization followed by precipitation heat treatment, the γ′-particles exhibited more irregular shapes after the (c) 2 h and (d) 30 min heat treatments. The γ-ligaments appeared to be wider for the water quenched sample after the 2 h heat treatment compared to the air cooled one. Furthermore, the amount of secondary γ′-particles was higher. The particle size of the sample heat treated for 30 min was in the range of the corresponding air cooled sample, but there were more irregularly shaped particles.

To quantify the differences in γ′-particle size and amount, the γ′-edge length and -volume fraction was measured. The results are summarized in Table 3 and reflect the significant difference in γ′-particle size between the precipitation heat treatment for 30 min (AC: 224 ± 52 nm; WQ: 231 ± 75 nm) and 2 h (AC: 324 ± 92 nm; WQ: 318 ± 108 nm). Regarding the γ′-volume fraction, the 2-h precipitation heat treatment shows a higher γ′-volume fraction (AC: 60.0 ± 0.4%; WQ: 59.0 ± 0.5%) than the samples heat treated for 30 min (AC: 58.3 ± 1.7%; WQ: 57.7 ± 1.5%). However, due to the calculated standard deviations, the difference is not significant.

All in all, the results show that a reduction of the γ′-particle size characterized by the γ′-edge length is possible, by reducing the holding time during precipitation heat treatment. The results indicate that the γ′-volume fraction is almost unchanged.

### 3.3. Reduction of the Precipitation Temperature

Reducing the precipitation temperature can have a number of effects. It not only slows down γ′-growth, so that smaller γ′-particles are expected for a given holding time, but may also influence phase fractions and the misfit δ. Here we explore these effects, starting with a precipitation heat treatment temperature of 1080 °C. Besides the standard heat treatment time of 6 h, a reduced duration of 4 h was also investigated.

**Figure 3:**
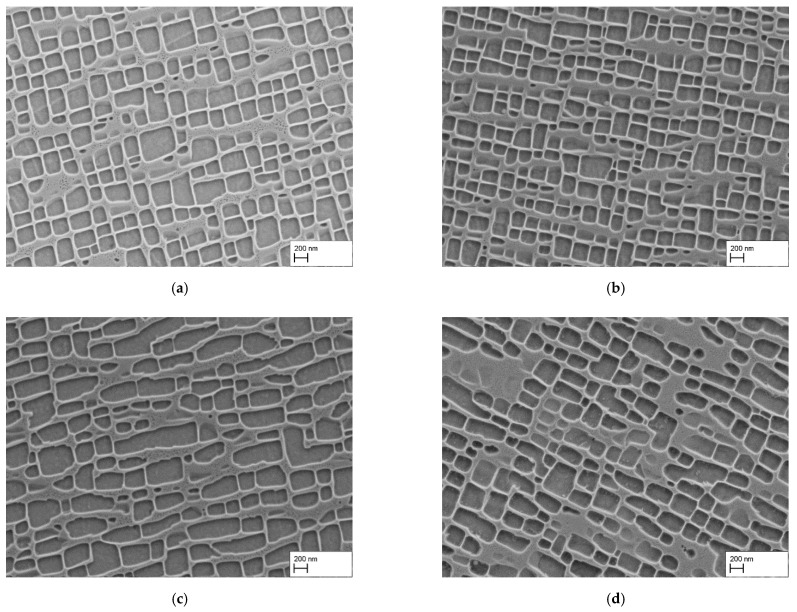
γ/γ′-microstructure after homogenization and precipitation heat treatments: (**a**) HT/AC + 1080 °C/6 h/AC, (**b**) HT/AC + 1080 °C/4 h/AC, (**c**) HT/WQ + 1080 °C/6 h/AC, (**d**) HT/WQ + 1080 °C/4 h/AC.

Figure 3a shows the microstructure of a heat treated sample at 1080 °C for 6 h with air cooling after homogenization heat treatment. The γ′-particles appear to be slightly smaller compared to the 1140 °C sample, while the alignment is still present. However, the corners of the γ′-particles are more rounded, so that the particles appear less edgy. Furthermore, the particle size varies, several bigger γ′-particles are present, sometimes with irregular shapes. The reduction of the holding time from 6 h to 4 h (b) resulted in smaller γ′-particles. Particles are well aligned, but the edges are still rounded. In the case of the water quenched sample, the 6 h heat treatment at 1080 °C in (c) resulted in irregular shaped γ′-precipitates, with mostly bigger sizes than the air cooled variant. The γ′-particles have an elongated shape, which indicates that the particles had grown together. This behavior was not present in the air cooled samples. The irregular shape is similarly present in the 4 h sample, as seen in (d), but not as pronounced as in (c).

The size reduction due to the reduced duration of the heat treatment at 1080 °C is also visible in the results of the γ′-edge length measurements in Table 4. For the AC samples the size reduced from 333 ± 109 nm to 271 ± 85 nm, while the γ′-volume fraction remained essentially constant. In the case of the water quenched samples, the size reduction was also confirmed by the measurements. Nevertheless, γ′-particle sizes were greater than the values of the air cooled samples (WQ: 6 h: 412 ± 171 nm and 4 h: 328 ± 113 nm), while maintaining nearly the same γ′-volume fraction (WQ: 6 h: 57.2 ± 3.3% and 4 h: 54.5 ± 2.1%). The qualitative microstructure examination and the γ′ measurements suggest that it is possible to reduce the γ′-particle size with both a diminished heat treatment temperature and heat treatment time.

In the context of this heat treatment study, we were also interested in exploring the effect on the microstructure when the diffusion distance $X\approx \sqrt{Dt}$ (*D*: Diffusion coefficient, *t*: time) was essentially kept identical to that at 1080 °C/4 h but the temperature was reduced significantly. For this purpose, heat treatments were conducted at 980 °C and 930 °C. Using

(1) $$D=D_{0}e^{\frac{-Q}{RT}},$$

with *D*_0_ = 2.32 × 10^−6^ m^2^ s^−1^, Q = 285 kJ/mol for self-diffusion of nickel [16,20], and *R* = 8.314 kg m^2^ s^−2^ mol^−1^ K^−1^, holding times of 30 h and 93 h were calculated for 980 °C and 930 °C, respectively.

**Figure 4:**
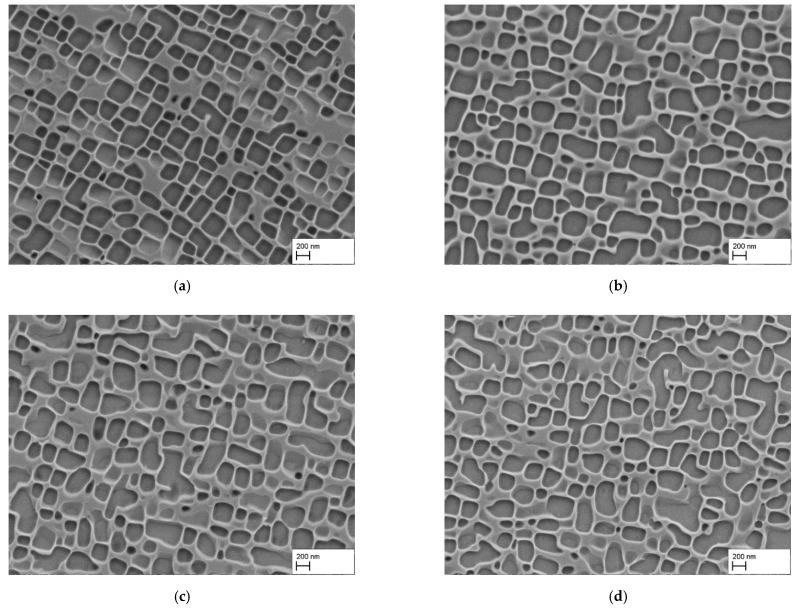
γ/γ′-microstructure after homogenization and precipitation heat treatments with precipitation times predicted by calculation: (**a**) HT/AC + 980 °C/30 h/AC, (**b**) HT/AC + 930 °C/93 h/AC, (**c**) HT/WQ + 980 °C/30 h/AC, (**d**) HT/WQ + 930 °C/93 h/AC.

The resulting microstructures are summarized in Figure 4a–d. By comparing the microstructures of Figure 4 with the ones from Figure 3b,d, it is apparent that only the microstructure of Figure 4a is similar. The other ones show obvious irregularities regarding the γ′-particle size and shape. Even, the γ′-particles in Figure 4a are more roundly shaped and the alignment is not as pronounced as in the sample heat treated at 1080 °C for 4 h. The results for the mean γ′-edge length and the γ′-volume fraction are summarized in Table 5.

Owing to the irregularity and the different forms and shapes in case of the samples heat treated at 930 °C for 93 h, a measurement of the γ′-edge length was not possible and meaningful. The results show that the γ′-volume fraction was higher for the AC sample (58.2 ± 0.5%) compared to the WQ sample (56.4 ± 0.7%). In comparison to the sample heat treated at 1080 °C for 4 h, the γ′-volume fraction only differed by about 1% (1080 °C/4 h/AC: 57.5 ± 2.0%). The γ′-edge length was also comparable. The AC sample heat treated at 1080 °C for 4 h showed a mean γ′-edge length of 271 ± 85 nm, while the AC sample heat treated at 980 °C for 30 h showed a value of 251 ± 100 nm. However, the mean γ′-edge length of the water quenched sample was about 311 ± 155 nm, which is in the range of the water quenched sample heat treated at 1080 °C/4 h (328 ± 113 nm).

## 4. Discussion

The results in this study demonstrate that there is a notable impact on the γ/γ′-microstructure and γ′-particle morphology when using water quenching instead of air cooling after homogenization heat treatment. As expected, water quenching leads to a significantly higher density of γ′-particles compared to air cooling in the as-quenched state (compare Figure 1a,d). This is a consequence of the higher undercooling, leading to a larger thermodynamic driving force for precipitation and, thus, a higher nucleation rate but less time for particle growth [17,21,22]. However, contrary to our expectations, water quenching did not result in particularly fine γ′-particles after precipitation heat treatment. Instead the results showed a pattern whereby WQ leads to larger γ′-edge lengths compared to AC. Furthermore, the particles were less regular in shape and alignment in the case of WQ, and coalescence of neighboring particles was frequently observed. To rationalize this finding, let us start with the situation after water quenching from the homogenization temperature. As mentioned, there is now a very high density of γ′-particles. As the particles are also very small, the interface energy, and not the elastic strain energy, is the decisive factor. Consequently, the coalescence of particles is energetically favorable, as it diminishes the total interface area. This happens early on during particle growth because the particles are very close to each other. In contrast, the particle density after AC is much smaller. Before the particles get close to each other during growth, the elastic strain energy becomes decisive. This has a number of important consequences. In order to reduce the elastic strain energy, the γ′-particles not only become cubic, they also align with each other along {001} side faces and repel each other, when they come close to each other [23]. All this does not happen to the same extent after WQ, due to the initially smaller size of the particles and distance between them. Of course, after WQ the γ′-particles also eventually become cubic as they grow, see e.g., Figure 3c. However, the initial lack of alignment and initial coalescence of particles are still visible after precipitation heat treatment. Coalesced particles lead to larger edge lengths. This explains the trend that water quenched samples generally exhibit larger γ′-edge lengths after precipitation heat treatment. The results clearly show that water quenching is counterproductive for fabrication of superalloy membranes with the finest possible pores. Note, however, that the same holds true if the cooling rate is too low. In fact, investigations in [8], on CMSX-4, showed that the γ′-particle size after precipitation heat treatment increases when the cooling rate is decreased from AC to 4 K/min to 1 K/min to 0.2 K/min. Thus, AC seems close to the optimum if a fine, well aligned γ/γ′-microstructure is the objective. Even though the focus here is on superalloy membranes, it is noteworthy that such a microstructure is also beneficial for high temperature applications, suggesting an optimal cooling rate from homogenization in this case as well.

The second factor investigated was the duration of the precipitation heat treatment. The results obtained at 1140 °C clearly demonstrated a significant effect. While heat treatment at 1140 °C/6 h led to a mean γ′-edge length of 345 nm in the case of AC after homogenization, the value decreased to 224 nm for the heat treatment at 1140 °C/30 min. Thus, reducing the precipitation heat treatment time is clearly a viable means to achieve a fine γ/γ′-microstructure. The γ′-particles are also well aligned and exhibit a pronounced cubic shape after this latter heat treatment, making it very attractive for the fabrication of superalloy membranes with a particularly fine and well-aligned porosity.

Finally, the precipitation heat treatment temperature was varied. As mentioned, this can have a number of effects on the microstructure evolution. Clearly, the most pronounced effect noticed here was on shape evolution. While the γ′-shapes after precipitation heat treatment at 930 °C were irregular and the corners of the particles were rounded, the particles became more and more cubic with increasing temperature. This is due to the changing misfit δ with temperature [14]. At ambient temperature, CMSX-4 exhibits a small, predominantly negative misfit [24], meaning that the lattice parameter of the γ-phase is slightly larger than that of the γ′-phase. As the thermal expansion coefficient of the γ-phase is larger than that of the γ′-phase, the misfit becomes more negative with increasing temperature [14]. Consequently, the stored elastic strain energy increases with temperature, favoring cubic shapes and well-aligned particles over rounded and irregularly arranged ones. This explains the observed morphological evolution of the γ′-phase. Note, that the shape of the γ′-particles not only depends on the misfit, but also on their size. However, the experiments at 930 °C and 980 °C were conducted in such a way that the diffusion distance $X\approx \sqrt{Dt\;}$ was equivalent to the heat treatment at 1080 °C/4 h and, consequently, the mean γ′-edge lengths at 980 °C and 1080 °C were similar (compare Table 5 with Table 4). Thus, the observed morphological differences can be clearly linked to the change in δ.

Besides experiments at constant diffusion distance X, the precipitation heat treatment temperature was also changed from 1140 °C to 1080 °C, with a constant duration of 6 h. After 1140 °C/6 h, the mean γ′-edge lengths were 345 nm and 445 nm for AC and WQ, respectively. After 1080 °C/6 h, values of 333 nm and 412 nm, respectively, were obtained. This shows the expected trend of decreasing γ′-particles size with decreasing temperature at a constant holding time, even though the observed effects were slight. With decreasing temperature, one would also expect the γ′-volume fraction to increase. However, the measured values did not show this trend. Inspecting the data where air cooling was used after precipitation heat treatments, all values are close to each other. At 1140 °C the measured mean γ′-volume fraction ranges from 57.3% to 60.0%. With one exception, the measured values at 1080 °C are close to 57%, and the same holds true for the results obtained at 980 °C and 930 °C. Using furnace cooling after 1140 °C led to significantly higher values of 64.4% to 69.1%. This is, in fact, expected because the supersaturation of the γ-matrix with γ′-forming elements during cooling can be reduced more completely with a decreasing cooling rate. However, for the same reason, a precipitation heat treatment at 980 °C or 930 °C should lead to a significantly higher γ′-volume fraction compared to heat treatment at 1140 °C, because solubility of the γ′-forming elements is considerably less at the former temperatures. In this context it is pointed out that the γ′-content varies within the microstructure, being less in the dendritic regions and more in the interdendritic ones [25]. As only three micrographs were taken per sample, these differences may have been averaged out incompletely. This qualification must be born in mind when considering these values.

Finally, we come back to the practical aspect of these investigations, namely to find a heat treatment procedure for superalloy membrane fabrication, leading to a porosity after selective phase extraction that is as fine and regular as possible. To enable this, the γ/γ′-microstructure after homogenization and precipitation heat treatment must be as fine and regular as possible. The important findings in this respect are, first, that air cooling from the homogenization treatment is ideal, as it facilitates good alignment of the γ′-particles and leads to particularly small particles for a given precipitation heat treatment. Second, in case of CMSX-4, it is not advisable to use low precipitation heat treatment temperatures, because the low misfit δ leads to irregular and poorly aligned γ′-particles. Instead, a high temperature in combination with a short duration should be used to achieve a fine, well-aligned γ/γ′-microstructure. Air cooling from homogenization in combination with precipitation heat treatment at 1140 °C/30 min followed these findings and led, in fact, to a particularly fine γ/γ′-microstructure, with a mean γ′-edge length of 224 ± 52 nm. We suggest using this heat treatment protocol if the aim is to produce CMSX-4 based superalloy membranes with particularly fine pores.

## 5. Conclusions

In this study, the effect of water quenching and the reduction of the precipitation temperature and time on the γ/γ′-microstructure of CMSX-4 was investigated for superalloy membrane development. The precipitation temperature was varied between 1140 °C and 930 °C, and the holding time during precipitation heat treatment between 30 min and 93 h. The following conclusions can be drawn from this investigation:

1. The use of water quenching (WQ) after homogenization heat treatment has a significant impact on the microstructure of CMSX-4 in the as-quenched state, resulting in a high density of comparatively small spherical γ′-precipitates.
2. Compared to air cooling (AC) after homogenization heat treatment (HT), water quenched samples often show irregular γ′-particle shapes and a less regular alignment of these particles after a subsequent precipitation heat treatment. This is attributed to particle coalescence in an early phase of particle growth. Thus, contrary to our expectations, WQ is counterproductive for the fabrication of superalloy membranes with small, well aligned porosity, while AC turned out to be favorable.
3. The γ′-morphology was studied for different precipitation temperatures at constant product D·t. In this way, the influence of the misfit δ could be observed at an essentially constant particle size. The results confirm a strong temperature dependence. While heat treatment at 1080 °C led to well-aligned, cubic precipitates, irregular particles with rounded corners resulted from heat treatments at 980 °C and 930 °C. This demonstrates that precipitation heat treatment temperatures below 1000 °C are not suitable for the fabrication of CMSX-4 based superalloy membranes with well-aligned porosity.
4. As expected, reducing the holding time during precipitation heat treatment restricts the growth of γ′-particles, resulting in smaller γ′-precipitates.
5. Based on these findings, HT/AC + 1140 °C/30 min/AC was identified as a promising heat treatment procedure for the purpose discussed here, as it leads to a particularly fine and well-aligned γ/γ′-microstructure.

## Figures and Tables

**Table 1:** Overview of the heat treatment steps and sample designation used in this study.

Heat Treatment	Temperatures and Times	
Homogenization heat treatment (HT)	1277 °C/2 h	
	1288 °C/3 h	
	1296 °C/3 h	
	1304 °C/2 h	
	1313 °C/2 h	
	1316 °C/2 h	
	1318 °C/2 h	
	1321 °C/2 h	
Cooling form homogenization	**AC** to room temperature	**WQ** to room temperature
Precipitation heat treatment	1140 °C/6 h/AC	
	1140 °C/6 h/FC 50 K/h until 550 °C reached	
	1140 °C/2 h/AC	
	1140 °C/30 min/AC	
	1080 °C/6 h/AC	
	1080 °C/4 h/AC	
	980 °C/30 h/AC	
	930 °C/93 h/AC	

**Table 2:** γ′-edge length and γ′-volume fraction of the AC and WQ samples with additional heat treatment at 1140 °C with subsequent AC or FC.

Heat Treatment	Point Analysis Results	Mean γ′-Volume Fraction	Standard Deviation	Mean γ′-Edge Length	Standard Deviation
HT/AC + 1140 °C/6 h	58.26%	59.6%	1.9%	345 nm	87 nm
	58.80%				
	61.80%				
HT/WQ + 1140 °C/6 h	55.40%	57.3%	2.3%	445 nm	182 nm
	56.66%				
	59.80%				
HT/AC + 1140 °C/6 h/50 K/h cooling	61.66%	64.4%	2.4%	370 nm	107 nm
	65.80%				
	65.66%				
HT/WQ + 1140 °C/6 h/50 K/h cooling	70.86%	69.1%	2.5%	566 nm	373 nm
	70.20%				
	66.20%				

**Table 3:** γ′-edge length and γ′-volume fraction of the AC and WQ samples with additional heat treatment at 1140 °C and a reduced precipitation time.

Heat Treatment	Point Analysis Results	Mean γ′-Volume Fraction	Standard Deviation	Mean γ′-Edge Length	Standard Deviation
HT/AC + 1140 °C/2 h	60.20%	60.0%	0.4%	324 nm	92 nm
	59.53%				
	60.33%				
HT/WQ + 1140 °C/2 h	59.60%	59.0%	0.5%	318 nm	108 nm
	58.93%				
	58.60%				
HT/AC + 1140 °C/30 min	58.86%	58.3%	1.7%	224 nm	52 nm
	56.33%				
	59.66%				
HT/WQ + 1140 °C/30 min	58.53%	57.7%	1.5%	231 nm	75 nm
	55.93%				
	58.66%				

**Table 4:** γ′-edge length and γ′-volume fraction of the AC and WQ samples with additional heat treatment at 1080 °C.

Heat Treatment	Point Analysis Results	Mean γ′-Volume Fraction	Standard Deviation	Mean γ′-Edge Length	Standard Deviation
HT/AC + 1080 °C/6 h	58.00%	58.0%	0.2%	333 nm	109 nm
	58.13%				
	57.73%				
HT/WQ + 1080 °C/6 h	55.06%	57.2%	3.3%	412 nm	171 nm
	61.00%				
	55.60%				
HT/AC + 1080 °C/4 h	55.00%	57.2%	2.0%	271 nm	85 nm
	58.73%				
	58.00%				
HT/WQ + 1080 °C/4 h	55.80%	54.5%	2.1%	328 nm	113 nm
	52.00%				
	55.60%				

**Table 5:** γ′-edge length and γ′-volume fraction of the AC and WQ samples with additional heat treatment at 980 °C and 930 °C.

Heat Treatment	Point Analysis Results	Mean γ′-Volume Fraction	Standard Deviation	Mean γ′-Edge Length	Standard Deviation
HT/AC + 980 °C/30 h	58.00%	58.2%	0.5%	251 nm	100 nm
	57.80%				
	58.73%				
HT/WQ + 980 °C/30 h	55.66%	56.4%	0.7%	311 nm	155 nm
	56.86%				
	56.73%				
HT/AC + 930 °C/93 h	55.13%	57.0%	2.0%	-	-
	58.93%				
	56.00%				
HT/WQ + 930 °C/93 h	56.33%	56.4%	0.1%	-	-
	56.26%				
	56.53%				

## Data Availability

Not applicable.
